# Intrathecal interleukin-6 levels are associated with progressive disease and clinical severity in multiple sclerosis

**DOI:** 10.1186/s12883-025-04145-0

**Published:** 2025-04-02

**Authors:** Justine Itorralba, Koroboshka Brand-Arzamendi, Georges Saab, Alexandra Muccilli, Raphael Schneider

**Affiliations:** 1 Department of Medicine, St. Michael’s Hospital, Unity Health Toronto, 30 Bond St, 17th floor, Peter Gilgan Tower, Toronto, ON M5B 1W8 Canada; 2 Faculty of Medicine, University of Toronto, Toronto, ON Canada; 3 University of Manitoba Multiple Sclerosis Clinic, Winnipeg, MB Canada

**Keywords:** Multiple sclerosis, Interleukin-6, Cerebrospinal fluid, Disease progression, Neuroinflammation

## Abstract

**Background:**

MS is characterized by persistent central nervous system (CNS) inflammation. Investigating the CNS-compartmentalized inflammation associated with progressive MS could uncover new biomarkers and therapeutic targets. Cerebrospinal fluid (CSF) interleukin-6 (IL-6) can be markedly elevated in neuroinflammatory conditions, such as neuromyelitis optica spectrum disorder and myelin oligodendrocyte glycoprotein antibody-associated disease. This study investigated the association between CSF IL-6 levels, progressive disease, and disease severity in MS.

**Methods:**

Advanced technologies, including single-molecule arrays and microfluidics, were used to analyse CSF samples from individuals with MS at the time of diagnosis for IL-6. IL-6 levels were then correlated with clinical course, disease severity, and other known biomarkers associated with inflammation and disease severity.

**Results:**

Elevated IL-6 levels in the CSF were found in individuals with progressive MS, and CSF IL-6 showed positive correlations with the Expanded Disability Status Scale, the Multiple Sclerosis Severity Score, and CSF glial fibrillary acidic protein levels.

**Conclusions:**

IL-6 in CSF indicates ongoing CNS inflammation and may contribute to the compartmentalized inflammation associated with disease progression and overall disease severity.

**Supplementary Information:**

The online version contains supplementary material available at 10.1186/s12883-025-04145-0.

## Background

The hallmark pathological features of MS are inflammation and neurodegeneration, which co-occur throughout the disease course [1]. Currently, people with MS (pwMS) are categorized into relapsing or progressive forms [2]; however, it has been suggested that progression recapitulates earlier, sometimes sub-clinic, relapses and exposes previous clinically silent lesions, indicating a complex overlap between relapsing and progressive disease that may be cooccurring in most if not all pwMS [3]. This overlap may explain heterogeneity in disease progression, treatment outcomes and prognosis between individual pwMS [4].

Biomarkers that reflect different aspects of the disease pathophysiology could be crucial in more accurately characterizing individuals, such as identifying those with features of more severe subclinical disease activity and individuals at high risk of accumulating disability. The potentiators of continued inflammation in progressive MS are thought to be CNS-compartmentalized within chronic active lesions and housed diffusely within the meninges, the choroid plexus, and concentrated ectopic lymph node follicles (eLFs) [5]. The formation and maintenance of eLFs involves a dynamic cytokine environment that supports the development and persistence of these structures, likely contributing to disease progression by fostering local immune cell interactions [6]. Understanding the CNS-compartmentalized inflammation in MS may yield opportunities to develop new biomarkers and therapies.

Neurofilament light chain protein (NfL) and glial fibrillary acidic protein (GFAP) are emerging biomarkers in MS that may predict disease progression [7, 8]. As both NfL and GFAP can be elevated in various neurological diseases [9, 10] without directly indicating inflammation, integrating inflammatory proteins such as cytokines into a biomarker panel could provide a more comprehensive assessment of the biology underlying MS. Despite some variability, previous work on cytokines in CSF of pwMS has shown several cytokines to be elevated rather consistently across studies, including interleukin IL-10 (IL-10) and tumour necrosis factor-alpha (TNF-α) [11]. The heterogeneities observed across studies may be attributed to different detection methods and challenges related to measuring proteins present only in low abundance in CSF. Using a highly sensitive single molecule array (SiMOA^®^), we recently found that granulocyte-macrophage colony-stimulating factor (GM-CSF) levels are higher in the CSF of pwMS compared to controls, correlating with other markers of intrathecal inflammation [12].

In this study, aiming to pinpoint intrathecal cytokines that could shed light on disease progression, we utilized cutting-edge protein detection methods, including SiMOA^®^ and microfluidics, to examine cytokines in the CSF of pwMS who were newly diagnosed with either relapsing-remitting (RRMS) or primary progressive MS (PPMS).

## Methods

This study was carried out in accordance with the World Medical Association’s Declaration of Helsinki for experiments involving humans. All individuals provided informed consent for the inclusion of their samples in the study (Unity Health Toronto REB#20–289). Samples were collected from 74 individuals undergoing diagnostic workups for multiple sclerosis at the outpatient clinic of St. Michael’s Hospital (BARLO MS Centre), Unity Health Toronto. No patients had developed new symptoms indicative of relapse or received steroids within the 90 days preceding the lumbar puncture. None had received disease-modifying therapy before or at the time of sample collection. All patients were eventually diagnosed with RRMS or PPMS according to the most recent diagnostic criteria [2]. Healthy control (HC) participants underwent a spinal tap to rule out a neuroinflammatory condition.

After collection, samples were immediately processed and stored at -80 °C following international standardization guidelines for the biobanking of CSF [13]. Samples were only thawed once, immediately prior to analysis. Routine clinical data available for all samples included age, sex, diagnosis, Expanded Disability Status Scale (EDSS) [14], and the Multiple Sclerosis Severity Score (MSSS) [15] at the time of CSF collection.

**Fig. 1:**
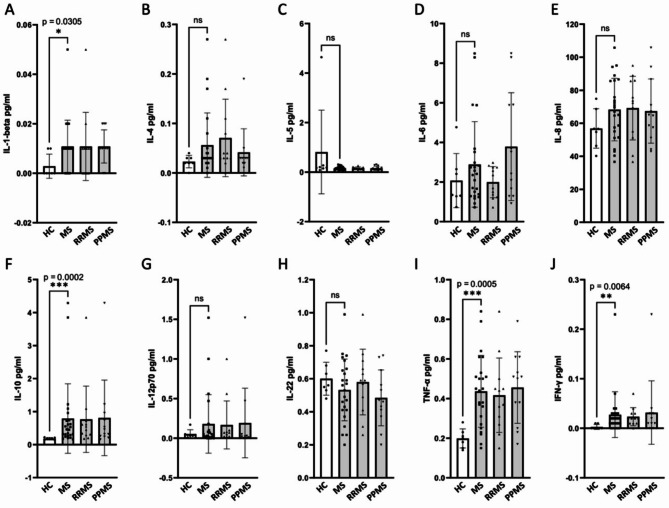
Cytokine levels in CSF of HCs vs. MS measured with SiMOA^®^ 10-PLEX Planar Array. **(A)** IL-1-beta levels were significantly higher in CSF from pwMS vs. HCs (*p* = 0.0305). **(B)** IL-4 levels were not significantly higher in CSF from pwMS vs. HCs. **(C)** IL-5 levels were not significantly higher in CSF from pwMS vs. HCs. **(D)** IL-6 levels were not significantly higher in CSF from pwMS vs. HCs. **(E)** IL-8 levels were not significantly higher in CSF from pwMS vs. HCs. **(F)** IL-10 levels were significantly higher in CSF from pwMS vs. HCs (*p* = 0.0002). **(G)** IL-12p70 levels were not significantly higher in CSF from pwMS vs. HCs. **(H)** IL-22 levels were not significantly higher in CSF from pwMS vs. HCs. **(I)** TNF-α levels were significantly higher in CSF from pwMS vs. HCs (*p* = 0.0005). **(J)** IFN-ɣ levels were significantly higher in CSF from pwMS vs. HCs (*p* = 0.0064). Mean with standard deviation (SD) are shown. Statistical significance was determined using the Mann-Whitney test. * *p* < 0.5, **p < 0.01, ***p < 0.001

Using the 10-PLEX SiMOA Planar Array^®^ technology (SP-X SiMOA^®^ platform, Quanterix, Billerica, MA, USA), we quantified ten CSF cytokines in the screening cohort (Table 1; Fig. 1). IL-6 was further quantified using Simple Plex Human NfL Cartridge with an Ella^®^ automated immunoassay system (Catalog # SPCKB-PS-003028, Protein Simple, San Jose, CA, USA, Table 1). CSF samples were diluted 1:4, and plasma samples were diluted 1:2, as per kit instructions, and concentrations were calculated from the corresponding standard curve. NfL was quantified in CSF using Simple Plex Human NfL Cartridge with an Ella^®^ automated immunoassay system (Catalog # SPCKB-PS-002448, Protein Simple, San Jose, CA, USA, Table 1). CSF samples were diluted (1:2) as per kit instructions, and concentrations were calculated from the corresponding standard curve. GFAP was quantified using Simple Plex Human GFAP Cartridge with the same platform (Catalog # SPCKB-HF-000970, Protein Simple, San Jose, CA, USA, Table 1). CSF samples were diluted (1:2) as per kit instructions, and concentrations were calculated from the corresponding standard curve. As per cartridge specifications, all samples were run in triplicate on an Ella^®^ Automated Immunoassay System (Bio-Techne, Minneapolis, MI, USA).

**Table 1:** Detection limits for the measurement of plasma proteins

Plasma proteins	Lower Limit of Quantification (LLOQ)	Upper Limit of Quantification (ULOQ)
*SiMOA Planar Array* ^®^		
Interleukin 1-beta (IL-1β)	0.02 pg/ml	100.00 pg/ml
Interleukin-4 (IL-4)	0.20 pg/ml	200.00 pg/ml
Interleukin-5 (IL-5)	0.05 pg/ml	200.00 pg/ml
Interleukin-6 (IL-6)	0.07 pg/ml	300.00 pg/ml
Interleukin-8 (IL-8)	0.10 pg/ml	400.00 pg/ml
Interleukin-10 (IL-10)	0.02 pg/ml	25.00 pg/ml
Interleukin 12, p70 subunit (IL-12p70)	0.07 pg/ml	300.00 pg/ml
Interleukin-22 (IL-22)	0.02 pg/ml	25.00 pg/ml
Interferon-gamma (IFN-ɣ)	0.01 pg/ml	50.00 pg/ml
Tumour Necrosis Factor-Alpha (TNF-α)	0.10 pg/ml	400.00 pg/ml
*Simple Plex Human NfL Cartridge with an Ella* ^®^ *Automated Immunoassay System*		
Interleukin-6 (IL-6)	0.28 pg/ml	2652 pg/ml
Neurofilament Light Chain (NfL)	2.70 pg/ml	10,290.00 pg/ml
Glial Fibrillary Acidic Protein (GFAP)	72.1 pg/ml	110,000 pg/ml

All statistical analyses and illustrations were performed in GraphPad Prism (v8). Not all data were normally distributed. The Mann-Whitney test was used to test for differences between groups, and Spearman’s rank coefficient was performed to test for correlations. Receiver operating characteristic (ROC) curves were calculated according to Wilson/Brown. Only p-values of < 0.05 were considered significant.

## Results

### Patient demographics and disability measures

CSF from participants in the screening cohort (Table 2) were included in the 10-PLEX SiMOA Planar Array^®^ (SP-X SiMOA^®^ platform). The majority of patients in both patient groups were female (62% of RRMS and 58% of PPMS). Patients with PPMS were older (mean age 49 years vs. 32 years, *p* < 0.001) and had a longer disease duration than those with RRMS (median 1 year vs. 3 years, *p* < 0.001), and higher EDSS (1 (range 0–4) vs. 4 (range 2-6.5) (*p* < 0.001)) (Table 2). CSF from participants in the complete cohort were included in the IL-6, NfL and GFAP Ella^®^ microfluidics experiments. The majority of patients in both patient groups of the complete cohort were female (65% of RRMS and 63% of PPMS). Patients with PPMS were older (mean age 40 years vs. 34 years, *p* < 0.001) and had a longer disease duration than those with RRMS (median 1 year vs. 3 years, *p* < 0.005), and higher EDSS (1 (range 0–4) vs. 4 (range 2-6.5) (*p* < 0.001)) (Table 2).

**Table 2:** Participant demographics and disability measures

**Screening cohort**				
	female/total (% female)	age in years mean (range)	disease duration in years median (range)	EDSS median (range)
HC	6/6 (100%)	44 (26–63)	n/a	n/a
RRMS	8/13 (62%)	32 (21–43)	1 (0–8)	1 (0–4)
PPMS	7/12 (58%%)	49 (29–69)	3 (1–29)	4 (2-6.5)
**Complete cohort**				
HC	8/9 (89%)	44 (26–65)	n/a	n/a
RRMS	32/49 (65%)	34 (19–55)	1 (0–15)	1 (0–4)
PPMS	10/16 (63%)	40 (29–69)	3 (1–12)	3.5 (2-6.5)

### Cytokine screening in CSF using the 10-PLEX SiMOA planar Array^®^

IL-1-beta, IL-10, TNF-α, and IFN-ɣ levels were significantly higher in CSF from pwMS, with p-values of 0.0305, 0.0002, 0.0005, and 0.0064, respectively (Fig. 1). Conversely, IL-4, IL-5, IL-6, IL-8, IL-12p70, and IL-22 levels did not show significant differences between the two groups. No significant differences were observed between the levels of any cytokine in individuals with RRMS vs. PPMS. However, IL-6 levels were numerically higher in PPMS than in RRMS (mean IL-6 in RRMS of 1.99 pg/ml in vs. 3.79 pg/ml in PPMS, Fig. 1D), prompting us to focus on IL-6 in subsequent experiments.

### Comparison and validation of CSF IL-6 quantification technologies

**Fig. 2:**
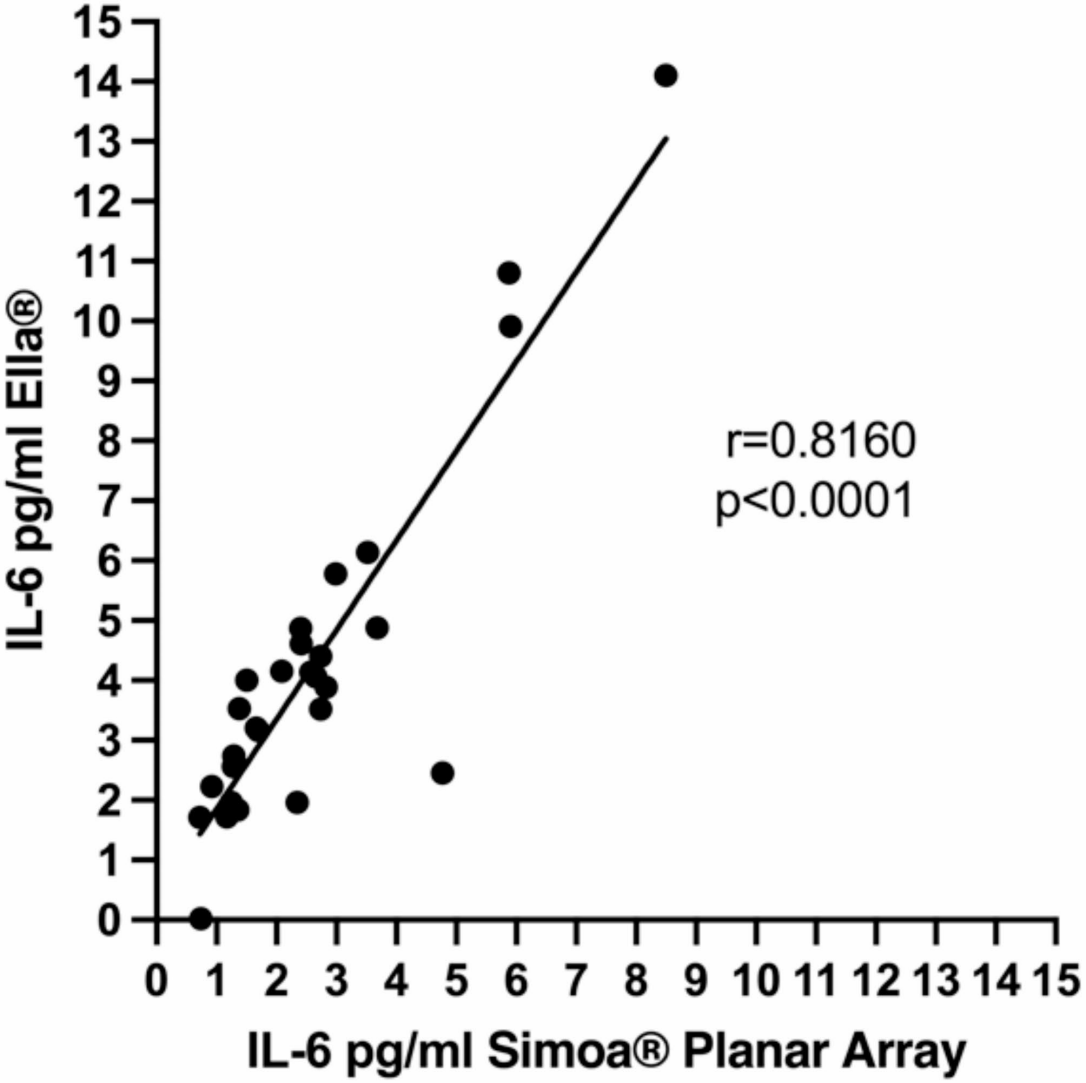
Ella vs. Planar Array. IL-6 levels in CSF were determined using the Ella^®^ microfluidics assay, which correlates with the SiMOA^®^ Planar Array results

We aimed to focus on CSF IL-6 using a reliable and cost-effective technology. To compare the CSF IL-6 values obtained from the 10-PLEX SiMOA^®^ Planar Array Technology with those from Ella^®^ microfluidics, we re-ran the available CSF samples from 30 out of 31 individuals from the screening cohort (96.8%) on the Ella^®^ platform. IL-6 levels showed a strong correlation with a Spearman correlation coefficient (r) of 0.8160 (*p* < 0.0001) (Fig. 2). Given that IL-6 was detectable in all individuals within the measurable range of Ella^®^ microfluidics and highly correlated with results from the 10-PLEX SiMOA^®^ Planar Array Technology, we selected the former technology to study the complete cohort (Table 2).

### Elevated CSF IL-6 levels and discriminative power in differentiating PPMS from RRMS

**Fig. 3:**
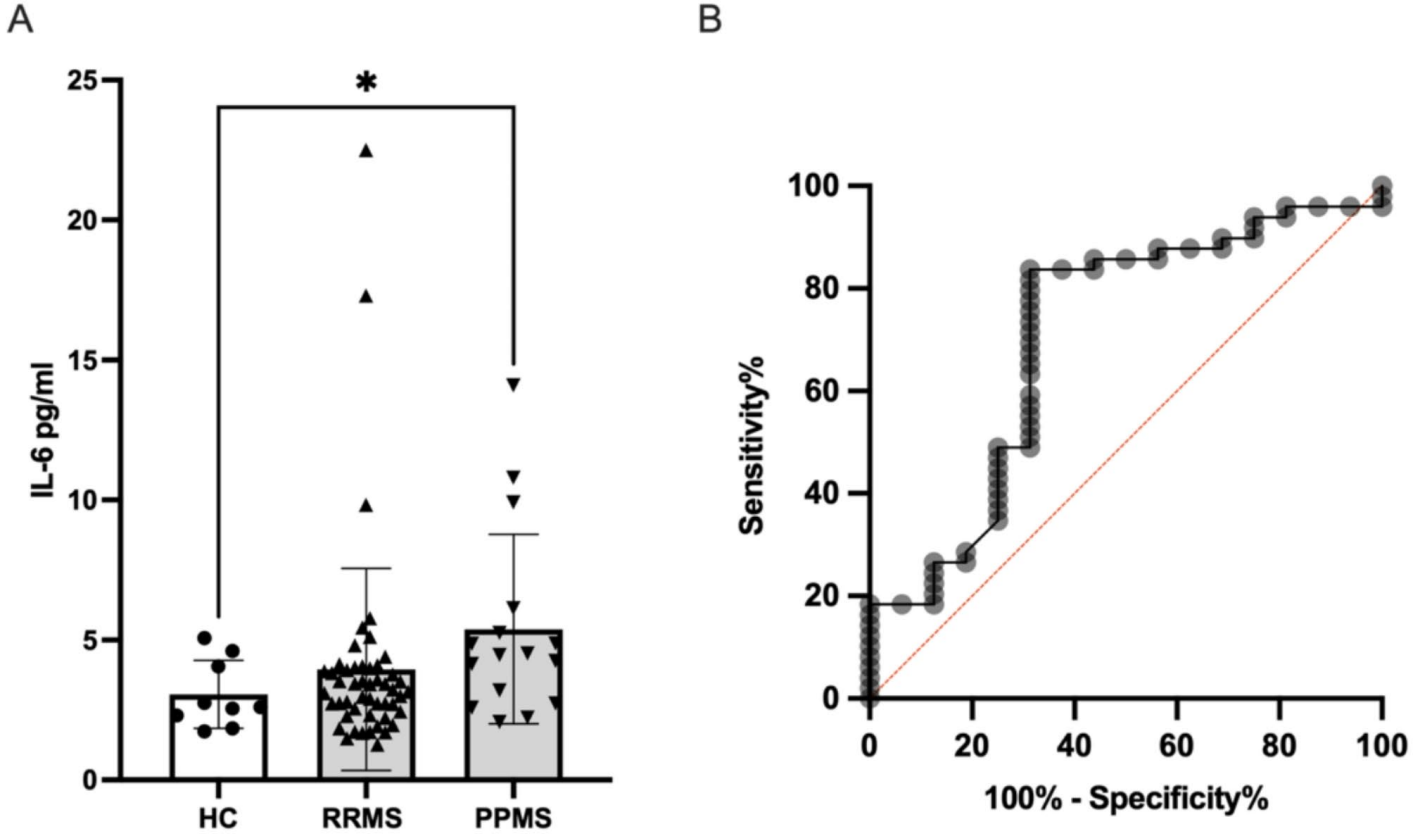
Intrathecal IL-6 is increased in PPMS. IL-6 levels in CSF of HCs vs. RRMS and PPMS measured with Ella^®^. (A) IL-6 levels were significantly higher in PPMS vs. HCs (*p* = 0.0428). Statistical significance was determined using the Mann-Whitney test. * *p* < 0.5. (B) Receiver operating characteristic (ROC) analysis demonstrated the capacity of IL-6 levels to distinguish between RRMS and PPMS with an area under the curve (AUC) of 0.7060, and statistical significance (*p* = 0.0139)

Using the Mann-Whitney test, we found a significant increase of IL-6 levels in the CSF of people PPMS vs. controls (*p* = 0.0428) (Fig. 3A). To determine whether age influenced the difference in CSF IL-6 levels between PPMS and controls, we conducted a Mann-Whitney U test on age-adjusted CSF IL-6 residuals. The difference remained statistically significant (*p* = 0.029, α = 0.05). Receiver operating characteristic (ROC) analyses aimed to determine the ability of intrathecal IL-6 levels to differentiate between RRMS and PPMS. We found that intrathecal IL-6 has a discriminative power with an area under the curve (AUC) of 0.7060, achieving statistical significance with a p-value of 0.0139 (Fig. 3B). People with PPMS were older than those with RRMS (Table 2). CSF IL-6 levels did not correlate with age when we analysed the entire cohort (*r* = 0.1453, *p* = 0.2168), as well as within individual groups, including controls (*r* = -0.5167, *p* = 0.1618), RRMS (*r* = 0.1553, *p* = 0.2868), and PPMS (*r* = 0.01325, *p* = 0.9628) (Supplementary Fig. 1A, B, C, and D). Mann-Whitney test was performed to compare CSF IL-6 levels between males and females, revealing no statistically significant difference between the sexes (*p* = 0.0959) (Supplementary Fig. 2).

### Intrathecal IL-6 correlates with EDSS and MSSS

**Fig. 4:**
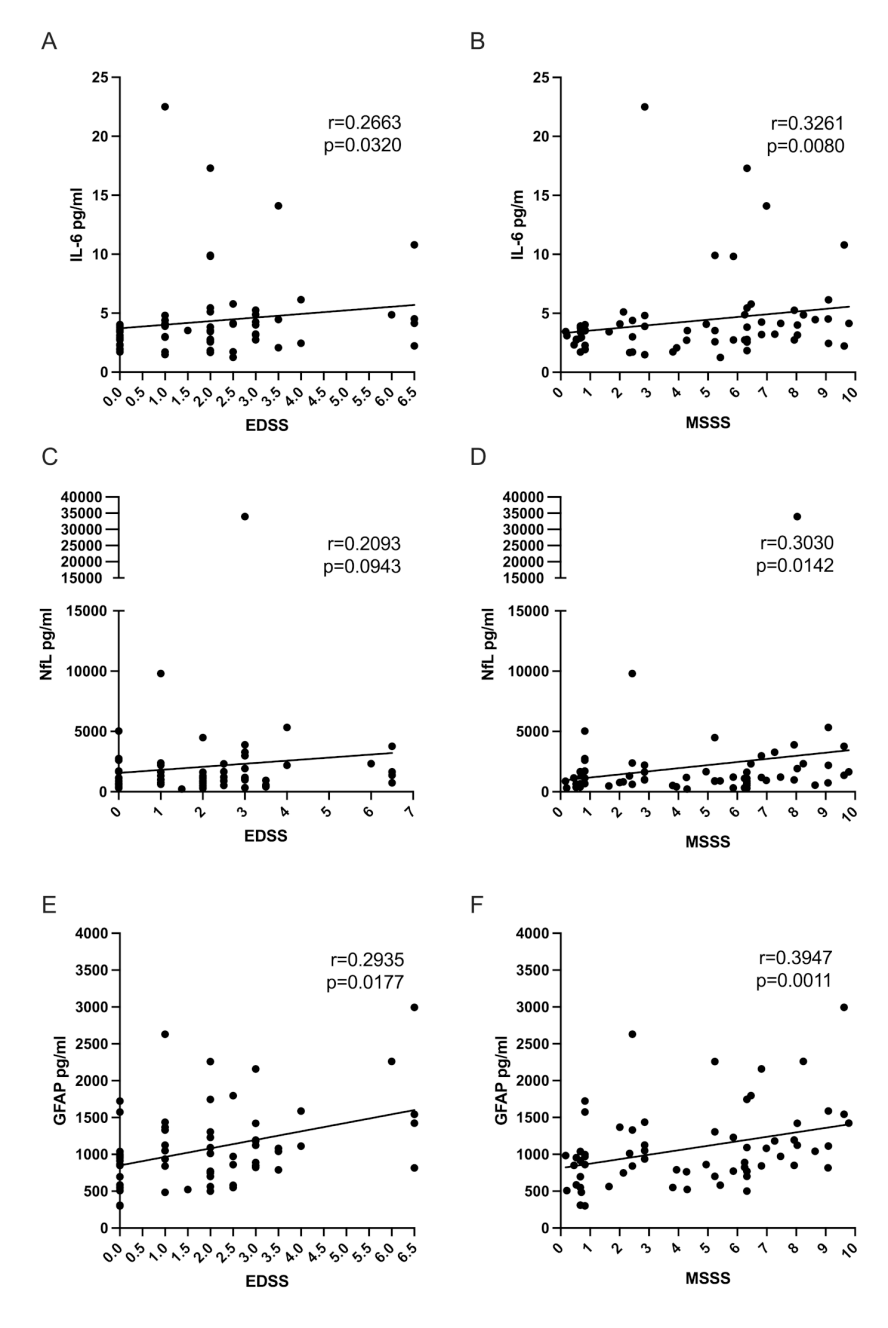
Correlation of intrathecal IL-6, NfL and GFAP with clinical scales. **(A)** IL-6 levels correlated with the EDSS, with a correlation coefficient (r) of 0.2663 and a p-value of 0.0320. **(B)** IL-6 levels also correlate with the MSSS, with *r* = 0.3261 and *p* = 0.0080. **(C)** NfL levels correlated with EDSS, albeit with a weaker correlation coefficient (*r* = 0.2093) and a non-significant p-value (*p* = 0.0943) **(D)** NfL levels correlated with MSSS, with *r* = 0.3030 and *p* = 0.0142. **(E)** GFAP levels in CSF correlate with EDSS, with *r* = 0.2935 and *p* = 0.0177. **(F)** GFAP levels correlated with MSSS, with *r* = 0.3947 and a highly significant p-value of 0.0011. All correlations were calculated using Spearman’s rank coefficient

We found no correlation between intrathecal IL-6 levels and patient age or disease duration (data not shown). IL-6 levels in CSF positively correlated with EDSS scores, *r* = 0.2663 (*p* = 0.0320, Fig. 4A). Furthermore, a stronger correlation was observed between IL-6 levels and the MSSS, with *r* = 0.3261 and a p-value of 0.0080 (Fig. 4B), suggesting IL-6 as a potential marker of disease severity in MS. The correlation of NfL levels in CSF with the EDSS was positive but weaker (*r* = 0.2093) and did not reach statistical significance (*p* = 0.0943) (Fig. 4C). NfL levels significantly correlated with the MSSS (*r* = 0.3030, *p* = 0.0142). GFAP levels in CSF correlated with the EDSS (*r* = 0.2935, *p* = 0.0177, Fig. 4D), and a stronger correlation was observed with the MSSS (*r* = 0.3947, *p* = 0.0011, Fig. 4E).

### Intrathecal IL-6 and GFAP levels correlate positively

**Fig. 5:**
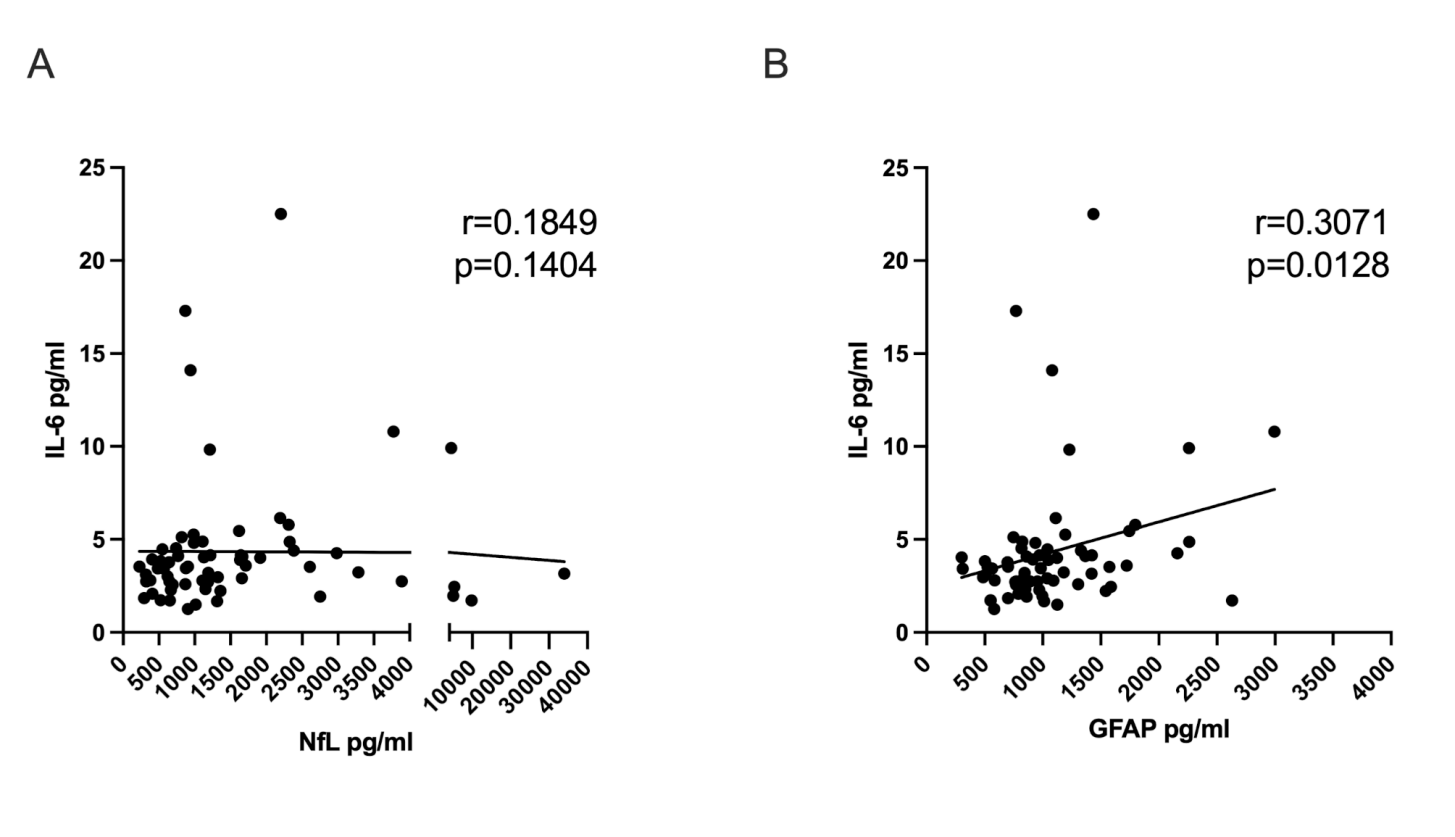
Correlation of intrathecal IL-6 with NfL and GFAP. **(A)** IL-6 levels did not significantly correlate with NfL **(B)** Increased IL-6 levels was associated with increased GFAP *r* = 0.3071 and *p* = 0.0128. All correlations were calculated using Spearman’s rank coefficient

Next, we examined the correlations between intrathecal IL-6 levels and two other relevant biomarkers that have been associated with disease severity and progressive disease, NfL and GFAP (Fig. 5). The analysis revealed distinct relationships with these biomarkers. Specifically, IL-6 levels did not significantly correlate with NfL, indicating that the changes in IL-6 levels within the intrathecal space do not parallel alterations in NfL concentrations (Fig. 5A). Conversely, we observed that increased IL-6 levels were associated with increased GFAP levels, with a Spearman’s rank correlation coefficient of 0.3071 and a p-value of 0.0128 (Fig. 5B). Additionally, IL-6 levels did not correlate with either CSF leukocyte count or CSF albumin (Supplementary Fig. 3A and B), suggesting that its elevation is not simply a reflection of generalized inflammation or blood-brain barrier dysfunction.

## Discussion

Our cytokine screening in CSF of pwMS using the 10-PLEX SiMOA Planar Array^®^ revealed that IL-1-beta, IL-10, TNF-α, and IFN-γ levels were significantly elevated in MS patients compared to healthy controls. However, IL-4, IL-5, IL-6, IL-8, IL-12p70, and IL-22 did not show significant differences when CSF levels from healthy control participants were compared to those from the entire MS cohort. Interestingly, IL-6 levels were numerically higher in PPMS than in RRMS, prompting further investigation of IL-6 as a marker of progressive disease. Given the detectability of IL-6 across all individuals within the measurable range of the Ella^®^ microfluidics and the strong correlation with the 10-PLEX SiMOA^®^ Planar Array Technology, we selected the Ella^®^ method for a wider study.

We demonstrated that IL-6 levels were significantly increased in PPMS and correlated with disease severity scales (EDSS and MSSS) in our MS cohort. Additionally, intrathecal IL-6 levels correlated with GFAP, but interestingly not with NfL. While research is still ongoing regarding the specific clinical uses of GFAP and NfL, the current understanding is that GFAP is a better marker for clinical progression, while NfL is better at predicting clinical relapses [7, 8]. The close relationship between IL-6 and GFAP may be partially explained by IL-6 expression in astrocytes and their dual association with astrocytic damage [16]. However, its specific role in progression rather than relapses may be related to the fact that IL-6 has been found on the leading edge of chronic active MS lesions [16], has been implicated in the formation of meningeal follicles [17], which have been postulated to underpin the smouldering damage that originates from the meninges, and has been postulated to be one of the key cytokines responsible for the shift in the overall inflammatory milieu from that of repair to progressive neurodegeneration [18].

Detecting intrathecal IL-6 using standard methods has previously been challenging. Data on potential correlations between CSF IL-6 levels and confounding factors such as age, sex, and weight remain limited. In our analyses, we did not find evidence that age or sex acted as confounding factors.

Bassi and colleagues investigated IL-6 in the CSF of pwMS and found that IL-6 was more frequently detectable in individuals with future disease activity, particularly in RRMS [19]. The differences between the Bassi study and our research might stem from technical factors, but it is also plausible that our results are complementary, as both studies identify IL-6 as a marker of progression within differently classified populations of pwMS, based on traditional RRMS versus PPMS categories.

In neuromyelitis optica spectrum disorder (NMOSD), intrathecal IL-6 and GFAP are elevated during attacks [20]. Further, increased IL-6 levels are associated with both NMOSD disease severity and risk of future relapse [21, 22]. Monoclonal antibody therapy targeting the IL-6-R has been shown to be effective in the treatment of NMOSD [23, 24], potentially by inducing regulatory B cells [25].

In myelin oligodendrocyte glycoprotein antibody-associated disease (MOGAD), CSF IL-6 levels are markedly increased in patients with brain lesions compared to those with spinal cord lesions [22]. Additionally, patients with elevated CSF IL-6 levels tend to experience poorer neurological recovery [22]. A recently reported case of fulminant MOGAD with extremely high CSF IL-6 levels and successful treatment using the IL-6-R blocker tocilizumab suggests that such therapies could be a viable option for similar cases [26].

While there has been a surge of new therapies targeted towards preventing clinical and radiographic relapses, treatments aimed at halting MS disease progression have been less successful. The results presented here suggest that similar to NMOSD and MOGAD, IL-6 may play a role in MS, emphasizing the relevance of IL-6 as a biomarker of disease severity and as a potentially critical player in disease pathogenesis and progression. The potential use of anti-IL6-R therapies for treating pwMS remains uncertain, as case report data indicate that these agents may cause or contribute to new lesion formation [27]. Of note, there are numerous potential alternative treatment approaches to interfere with IL-6 or its signalling pathways [28]. Moreover, Devasahayam et al. demonstrated that during maximal exercise, MS patients exhibited lower levels of IL-6 [29], suggesting that reducing IL-6 or its effects may be possible through methods other than disease-modifying therapy.

This study has limitations due to its retrospective nature, an imbalance in group sizes, and potential confounding factors. While CSF IL-6 levels were similar between the sexes and did not correlate with age in this study, larger prospective studies should consider these and other potential confounders to further evaluate the role of CSF IL-6 in disease progression. Nevertheless, our findings demonstrate significant correlations between elevated CSF IL-6 levels and clinical and laboratory indicators of disease severity, supporting IL-6 as a potential future biomarker for progressive disease pathology.

## Electronic supplementary material

Below is the link to the electronic supplementary material.

Supplementary Material 1

Supplementary Material 2

Supplementary Material 3

Supplementary Material 4

## Data Availability

Anonymized data are available to qualified researchers upon reasonable request.
