# Supplementary figures and images for: Intrathecal interleukin-6 levels are associated with progressive disease and clinical severity in multiple sclerosis
Source: BMC Neurol. 2025 Apr 2;25:136. doi: 10.1186/s12883-025-04145-0 (PMC11963510; doi:10.1186/s12883-025-04145-0)

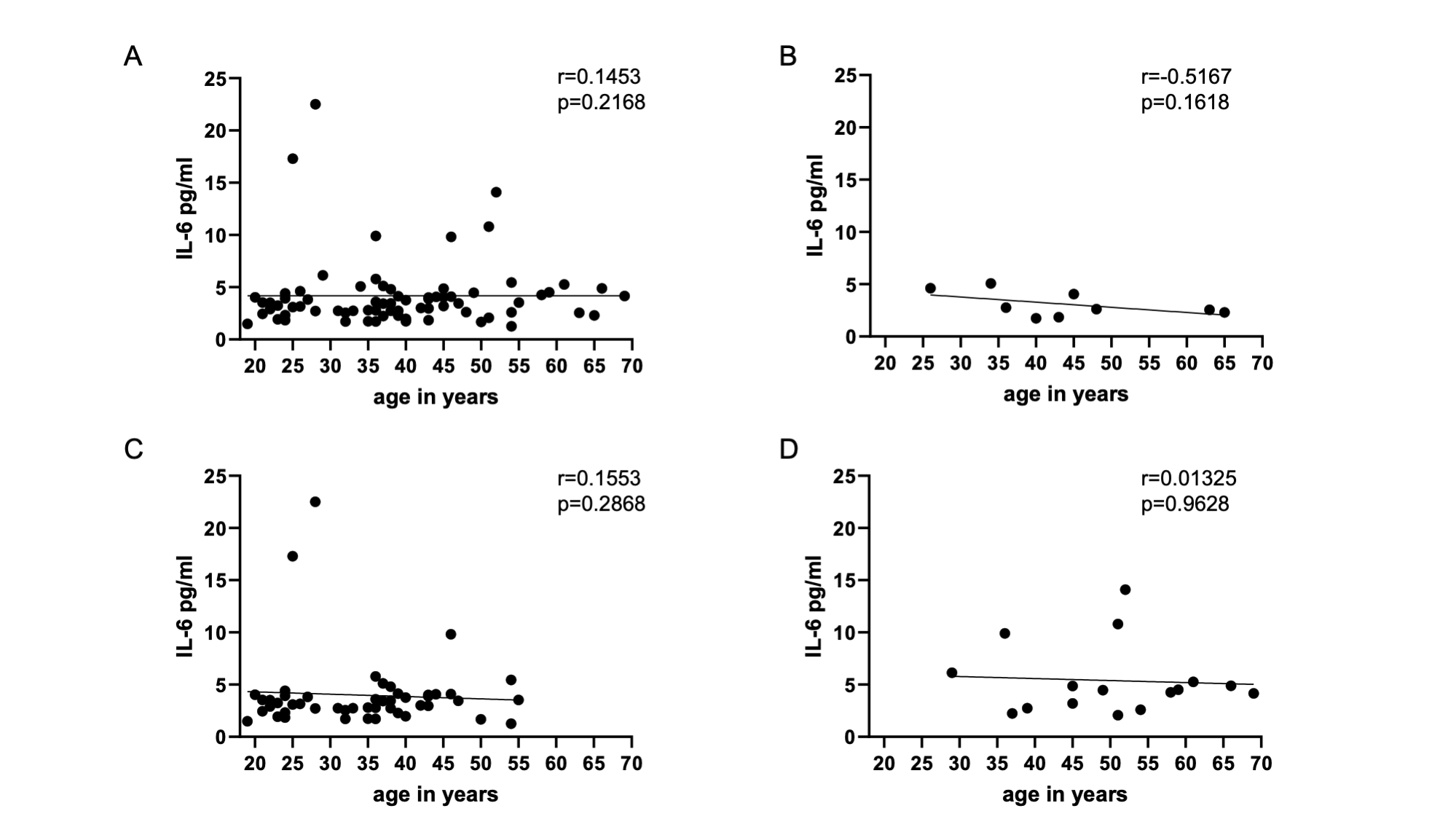

Supplement: Supplementary file 1 — Supplementary Material 1 [file 12883_2025_4145_MOESM1_ESM.tiff]

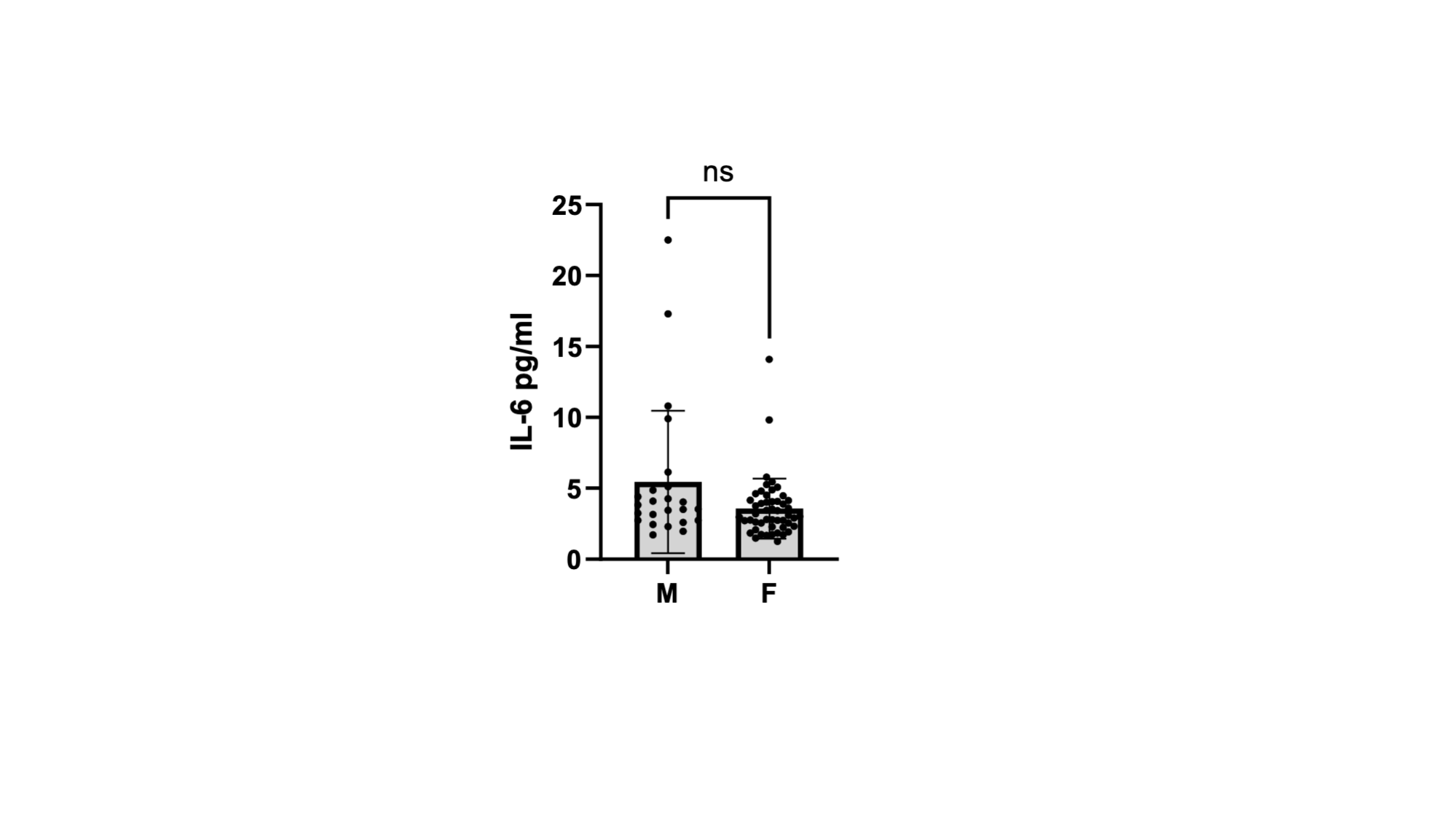

Supplement: Supplementary file 2 — Supplementary Material 2 [file 12883_2025_4145_MOESM2_ESM.tiff]

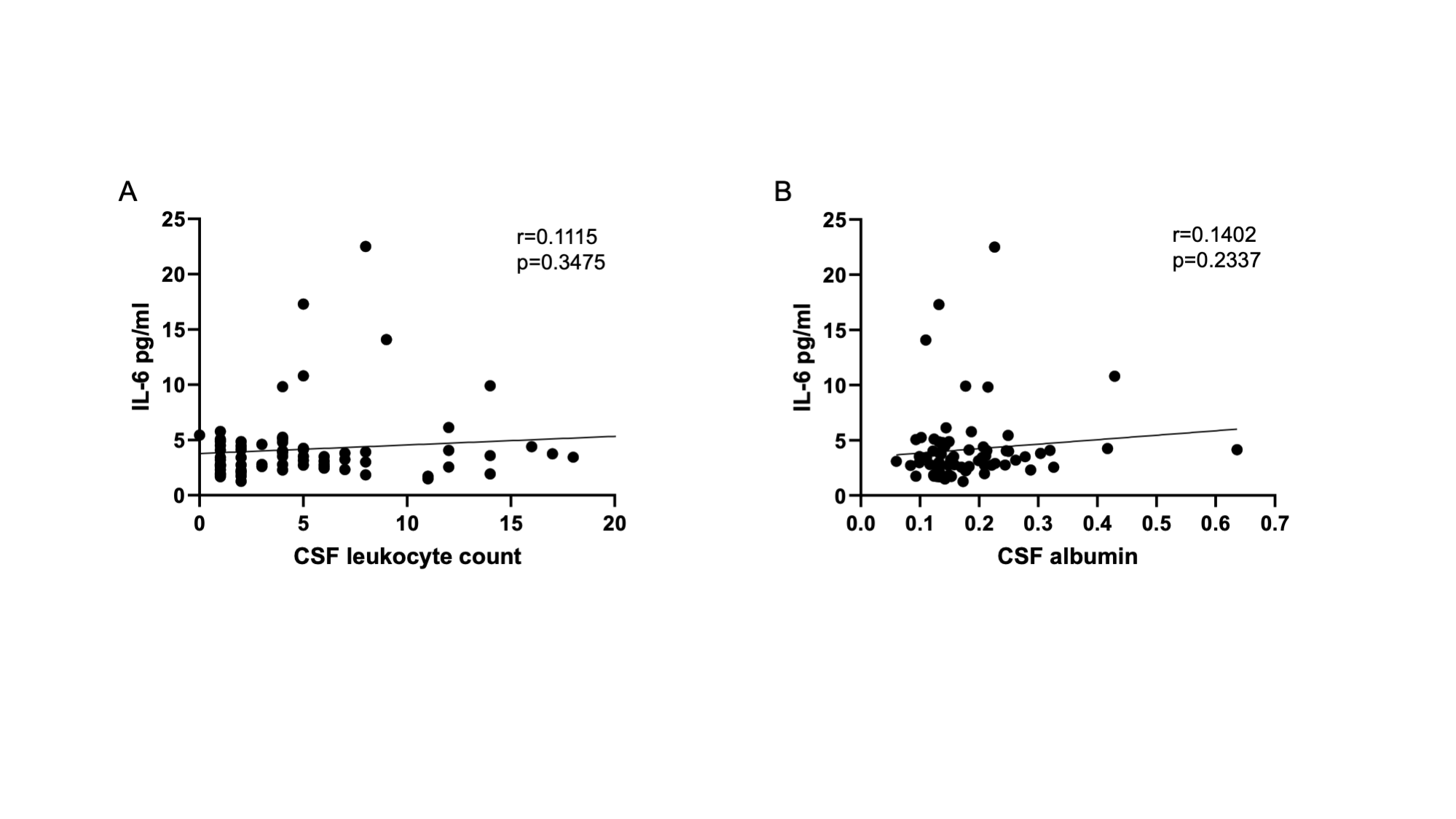

Supplement: Supplementary file 3 — Supplementary Material 3 [file 12883_2025_4145_MOESM3_ESM.tiff]
