# Supplementary material for: Intrathecal interleukin-6 levels are associated with progressive disease and clinical severity in multiple sclerosis
Source: BMC Neurol. 2025 Apr 2;25:136. doi: 10.1186/s12883-025-04145-0 (PMC11963510; doi:10.1186/s12883-025-04145-0)
Supplement: Supplementary file 4 — Supplementary Material 4 [file 12883_2025_4145_MOESM4_ESM.docx]

**Supplementary Figure 1. Correlation of CSF IL-6 levels with age. A.** We found no significant association between CSF IL-6 levels and age in the overall cohort (p = 0.2168), **B.** controls (p = 0.1618), **C.** RRMS (p = 0.2868), or **D.** PPMS (p = 0.9628). All correlations were calculated using Spearman’s rank coefficient.

**Supplementary Figure 2. CSF IL-6 levels do not differ between sexes.** Intrathecal IL-6 levels were compared between males (M) and females (F) using the Mann-Whitney test, revealing no statistically significant difference (p = 0.0959). Not significant (ns).

**Supplementary Figure 3. Correlation of intrathecal IL-6 with CSF markers of inflammation. A.** IL-6 levels showed no significant correlation with CSF leukocyte count (r = 0.1115, p = 0.3475). **B.** IL-6 levels showed no significant correlation with CSF albumin (r = 0.1402, p = 0.2337). All correlations were calculated using Spearman’s rank coefficient.
